# Coarse matching was sufficient to capture attention by working memory representations unless matching features with the target

**DOI:** 10.1186/s40359-025-02522-5

**Published:** 2025-03-03

**Authors:** Cenlou Hu, Ziwen Luo, Sai Huang, Bao Zhang

**Affiliations:** 1 Department of Psychology, Guangzhou University, Guangzhou, 510006 China; 2 Changde Vocational Technical College, Changde, 415000 China; 3 Department of Special Education, Guangzhou University, Guangzhou, 510006 China

**Keywords:** Perceptual similarity, Memory-driven attentional capture, Working memory, Task-relevance

## Abstract

**Background:**

In most theoretical frameworks, the effectiveness of attentional selection relies significantly on the perceptual similarity between the target template and visual input. Nevertheless, ambiguity exists surrounding whether attentional capture triggered by irrelevant representations in Working Memory (WM) is influenced by the perceptual similarity levels of features between WM content and its matching distractors.

**Methods:**

We designed a hybrid WM and visual search task, varying such perceptual similarity of colors across three levels: exact, high-similar, and low-similar matching. To quantify the extent of the capture effect, we compared these conditions against a neutral baseline (i.e., completely different color) using eye movement and behavioral data in two experiments.

**Results:**

We consistently observed robust attentional capture effects across two experiments, evident in both eye movement indices and manual reaction times. In Experiment 1, where WM representations solely matched features to visual search distractors (task-irrelevant scenario), we found that changes in perceptual similarity did not influence attentional capture. Conversely, in Experiment 2, where WM representations had the potential to match the visual search target (task-relevant scenario), we observed a significantly more robust attentional capture effect for high-similar matching compared to low-similar matching conditions.

**Conclusions:**

These findings imply that coarse matching between distractors and WM contents is sufficient to capture attention, unless the matching features potentially correspond to the visual target. Furthermore, task relevance sharpens perceptual sensitivity to visual input, highlighting distinct mechanisms underlying attentional capture by irrelevant representations and target templates within WM.

**Supplementary Information:**

The online version contains supplementary material available at 10.1186/s40359-025-02522-5.

## Background

Within a visually complex environment brimming with extensive visual information, visual attention proactively filters out irrelevant stimuli while selectively directing focus toward relevant stimuli. During this attentional selection process, working memory (WM) representations play a pivotal role in biasing perceptual processing towards stimuli that share features with the contents of WM [1, 2]. For instance, the target template or attentional template [3, 4, 5, 6], which represents a representation of the visual search target in WM, promotes selective attention towards the template-similar objects and facilitates target recognition [7].

The likelihood of attention bias to an object is determined by the extent of perceptual similarity it shares with the target template [2, 8, 9]. Higher precision target templates lead to more effective attentional guidance and faster search performance [10]. However, recent studies have contested the traditional view that attentional guidance relies on a complete and precious target template [11, 12]. They argue that early and rapid attentional guidance may involve a less precious or “fuzzier” template, differing from the level of precision necessary for definitive target decisions. This highlights the flexible and adaptive nature of attention, as it can prioritize relevant information without perfect fidelity to the stored memory representations [12].

Moreover, besides guiding attention toward objects resembling the target template, evidence indicated that attention can also be biased toward objects sharing features with irrelevant representations in WM, even when these memory-matched objects consistently serve as distractors rather than targets [13, 14, 15, 16, 17, 18, 19]. Although such memory-driven attentional capture has been widely observed in various experimental settings, our understanding of how the perceptual similarity between irrelevant WM representations and its feature-matched distractors influences this phenomenon remains inadequate. The sensory recruitment account suggests that shared neuronal resources in sensory areas contribute to bidirectional interference in the context of attention and WM [20, 21, 22]. Therefore, during visual search, the concurrent activation of sensory processing for both the memorized item and its matched incoming information augments the ability of WM representations to capture attention. This implied a direct, one-to-one relationship between the internal WM representation and its corresponding external perceptual representation. However, less direct evidence demonstrated that perceptual similarity between these two representations, especially when the external representation functioned as a distractor. Addressing this issue was pivotal in elucidating the underlying mechanisms through which internal WM contents exert an influence on our unintentional attention.

To date, only a limited number of studies have directly addressed this topic. Teng and Kravitz [23] examined the effects of perceptual similarity by systematically adjusting the color distance (0°, 15°, 35°, 55° and 75° in the color wheel scale based on the CIE Lab* color space) between the distracting singleton and the WM item. They revealed that attentional capture was significantly greater at distance of 15° and 35° compared to 75°. Moreover, similar results were observed when manipulating orientation as the matching attribute. These results suggested that higher similarity between the memorized item and the singleton distractor yielded stronger attentional capture. Using a similar manipulation of perceptual similarity with a color wheel scale, Kiyonaga and Egner [24] varied the angular distance between memorized item and target. They observed a non-linear pattern of attentional benefits: target search efficiency decreased at 10° and 20° memory-target distances compared to 0°, increased at 30°, and then decreased again from 30° to 60°. Kiyonaga and Egner [24] attributed the reduced bias towards targets at 10° and 20° from the memorized color to a suppression of similar information in feature space near WM items, which facilitates WM maintenance.

Despite some discrepancies in the results of these two studies, a similar trend was observable that both targets [24] and distractors [23], when possessing features more similar to memory representations, are more likely to bias attention. However, it is crucial to emphasize that our primary focus in this study is on attentional capture, defined as the involuntary shift of attention triggered by a salient stimuli inconsistent with the current goals [25]. Therefore, Kiyonaga and Egner [24] did not offer definitive evidence regarding our specific concern, as their research primarily centered on memory-target similarity. However, it is plausible that when a memorized item potentially matches a target feature (i.e., task-relevant scenario), could alter the pattern of attentional capture by memory-matched distractors [14, 26, 27]. In align with the current study, Teng and Kravitz [23] investigated the effect of the perceptual similarity between memory items and distractors on memory-driven attentional capture, however, we considered their conclusion to remain uncertain because its primary reliance on the comparison between conditions with memory-distractor distances of 15° or 35° versus 75°. Generally, colors separated by more than 30° in the CIE Lab color space are perceived as distinctly different [28]. Therefore, it is reasonable to assume that the observed differences in their study were primarily due to the contrast between two conditions with perceptually dissimilar WM representations, rather than reflecting a range of perceptual similarities among them.

The precision of WM representation is a crucial factor closely linked to perceptual similarity. WM representation maintained with a higher level of precision usually increased the levels of perceptual similarity between memory items and memory-matched information. Several researchers have found that the WM representation could capture attention regardless of the quality of the WM representations [16, 29, 30]. However, the study conducted by Williams et al. [19] reported the opposite result, that the capture effect was enhanced by more precious WM representation. One crucial factor that might contribute to this discrepancy is the factor of task relevance, which has been proven to modulate memory-driven attentional capture [14, 26, 27]. In those studies that failed to observe the effect of WM precision [16, 29, 30], the WM representation never matched features to the visual search target but only to distractors, which was considered a task-irrelevant scenario. However, the WM representation in Williams et al. [19] potentially matched either the distractors or the target, thus representing a task-relevant scenario. It is conceivable that the influence of WM precision on attentional capture in Williams et al. [19] originates from the ability of more precise representations to expedite accurate target recognition in task-relevant scenarios. In contrast, when the WM representation lacks the chance to match the target, the negligible effect of precision on attentional capture might stem from the likelihood of attention being drawn to a memory-matched distractor despite the coarse quality of the WM representation. Under such circumstances, it is logical to postulate that attentional capture is primarily determined by a coarse match between the memory representation and the distractor rather than a precise match unless the WM representation becomes task-relevant. This was the primary hypothesis under investigation in the current study.

In summary, the current study aimed to investigate the impact of perceptual similarity in memory-distractor matching on memory-driven attentional capture. Firstly, it sought to determine whether fine or coarse memory-distractor matching was necessary for the occurrence of this capture by assessing the effect of perceptual similarity. Secondly, it explored whether the effect of perceptual similarity on memory-driven attentional capture was influenced by task relevance. Crucially, to mitigate potential confusion stemming from significant differences that could yield disparate perceptions, as observed in Teng and Kravitz [23], we deliberately confined the manipulation of similarity levels within a narrow spectrum based on the CIE Lab color space. Three distinct levels of perceptual similarity—exact matching, high-similar matching, and low-similar matching—were included and compared with neutral condition to quantify the attention capture effect. In the exact matching condition, the matching color precisely mirrored the memorized color. Considering that mental representations in WM vary for each physical color on the color wheel, we define the high-similar matching color in the high-similar condition as the average deviation for each color, calculated from our previous research with 33 participants and 21,121 trials (mean = 9.56°, standard deviation = 8.01°). Correspondingly, in the low-similar matching condition, the matching color was defined as the color that deviated by one standard deviation from this average deviation.

Two experiments separately investigated the influence of perceptual similarity on memory-driven attentional capture in task-irrelevant scenarios (Experiment 1) and task-relevant scenarios (Experiment 2). If a precise matching mechanism is necessary for attentional capture, we anticipated greater capture effects at higher levels of perceptual similarity. Conversely, if memory-driven attentional capture operates through a coarse matching mechanism, we expected no significant differences across the three levels of perceptual similarity. According to our beforementioned speculation based on previous studies, we hypothesized that the memory-driven attentional capture operates with a coarse matching mechanism under task-irrelevant scenarios. In contrast, in task-relevant scenarios, the pattern of attentional bias could potentially vary, as suggested by previous research such as Woodman and Luck [26]. Typically, a fine-matched target that aligns closely with the attentional template can improve its discriminability, although the attentional benefits often exhibit a non-linear pattern as the level of perceptual similarity between the memory and target matching varies [24]. Therefore, we hypothesized that in a scenario where the WM representation has the opportunity to match the visual search target, it would facilitate the reliance on a fine-matching mechanism in Experiment 2. Consequently, this would result in an impact of perceptual similarity on memory-driven attentional capture.

## Experiment 1

### Methods

#### Participants

Studies examining memory-driven attentional capture through ocular measurements with within-subject designs had sample sizes ranging from 11 to 16 in prior research [31, 32, 33]. Conservatively, 21 participants were recruited in Experiment 1 (4 males aged 18–22; *M*_age_ = 19.19). All participants had normal or corrected-to-normal visual acuity and normal color vision. They signed an informed consent and were paid ¥ 40 for their time. All procedures were approved by the local ethics committee in accordance with the Declaration of Helsinki.

#### Stimulus, apparatus, and procedure

The experiment was conducted in a sound-attenuated and dimly lit room. All visual stimuli were presented with a black background on LCD monitor (resolution: 1024 × 768; frame rate: 85 Hz) about 57 cm away from viewers.

To gain more detailed insights into the temporal dynamics of processing in memory-driven attentional capture, eye movements during the visual search were recorded from the participant’s right eye using an Eye-Link 1000 plus (1000-Hz temporal resolution) infrared head-mounted eye tracker (SR Research Ltd., Mississauga, Ontario, Canada). An adjustable chin rest was used to maintain a stable head position. Participants underwent a standard nine-point grid calibration at the beginning of each block.

The memory and visual search stimuli were randomly selected from a master set of 180 evenly distributed on a circle of CIE Lab color space [34]. The memory items were presented as 1.5° × 1.5° colored squares on the central screen. The visual search stimuli consisted of four colored squares, each with a minimum distance of 72° on the color wheel to ensure perceptual differences. These squares were thickened to 0.15° and had two gaps on the center of the left and right sides. The gap width of the three distractors was 0.5°. The search target was unique, with one side having a small gap (0.5°) and the other having a large gap (1.0°). These four search stimuli were arranged around an imaginary circle with a radius of 6°, presented at either the 1, 4, 7, 10, or 2, 5, 8, or 11 o’clock positions.

**Fig. 1:**
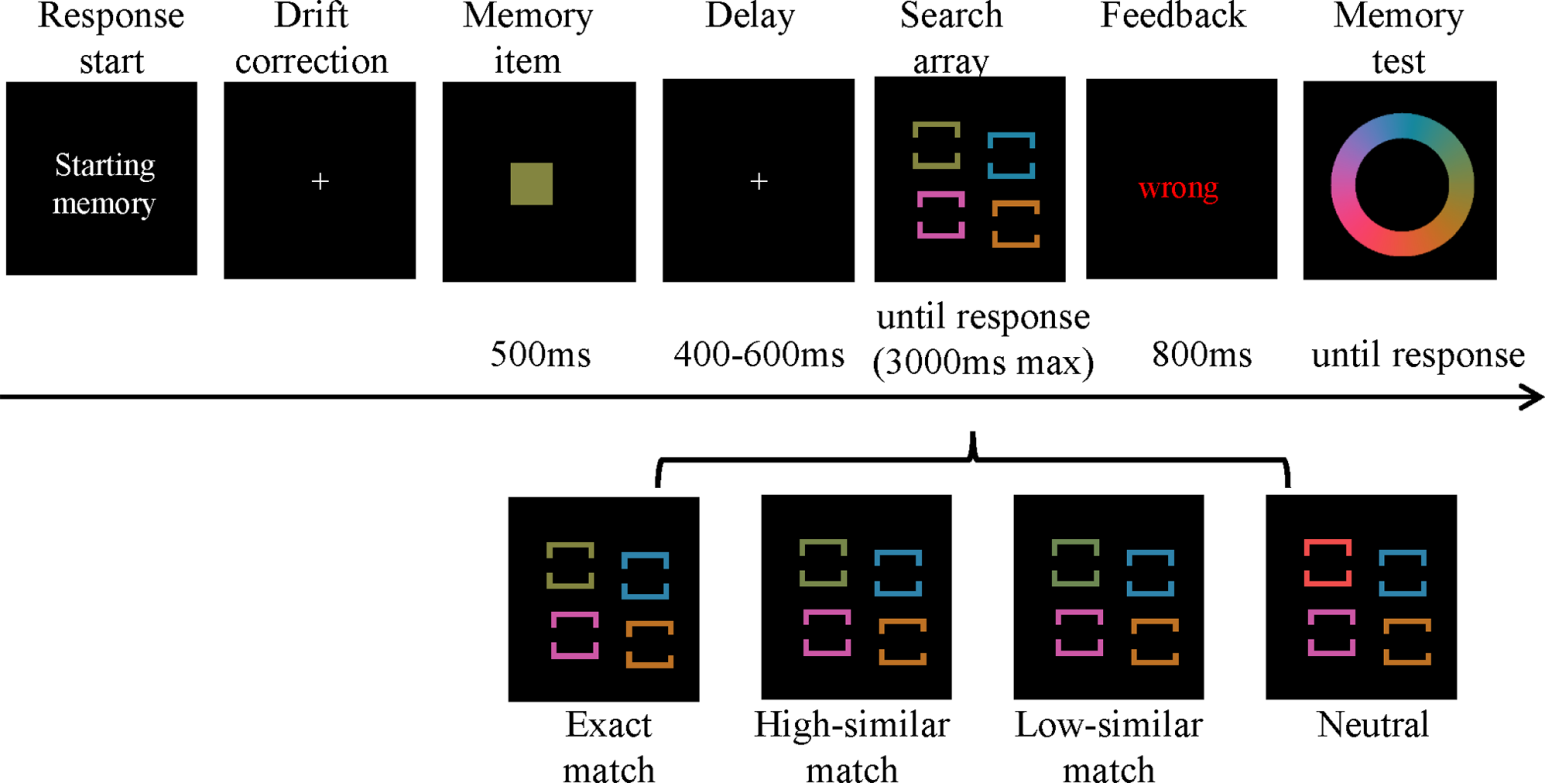
Schematic illustration of the event sequence in Experiment 1. Each trial begins with a Drift correction for eye movement once the participants voluntarily press a key to initiate the trial. After the drift correction, participants are instructed to memorize the color of a presented memory item and subsequently report the color by clicking on a color wheel with the mouse key during the final memory test. During the retention interval, a visual search display appears, where participants are required to search for the target square characterized by different gap sizes. Additionally, the figure depicts four distinct types of visual search displays employed in the experiment

As depicted in Fig. 1, each trial began with a reminder stating, “Starting memory”. This was followed by a fixation point at the center of the screen, which was used for drift correction. Once participants were detected as fixating on the fixation point for more than 1000 ms, the memory item was presented at the center of the screen for 500 ms. After a variable Inter Stimulus Interval (ISI) ranging from 400 ms to 600 ms, the visual search display appeared. In the visual search task, participants had to judge the side of the large gap quickly and accurately in the search targets within a maximum of 3000 ms. Feedback with text of “wrong” would appear for 800 ms if participants respond incorrectly. Finally, a color wheel was presented at the center of the screen. Participants were instructed to report the memorized color by clicking the left mouse button on the color wheel without time pressure.

In the visual search task, the color of a specific distractor was strategically manipulated across three varying conditions of perceptual similarity: exact matching, high-similar matching, and low-similar matching, as detailed in the introduction. To estimate attentional capture, a neutral condition was established as a baseline, where all colors of the visual search items differed by at least 72° from the memorized color. Furthermore, one of the distractors was randomly selected by the experimental program to serve as a contrast in each trial, enabling a comparative analysis of its ocular capture effect against the memory-matched distractor.

Each participant began with 24 practice trials to familiarize themselves with the task. Then, they completed three formal blocks, each corresponding to a distinct perceptual similarity block: exact, high-similar, and low-similar matching. Each block was randomly mixed with 72 trials for the memory-matching condition and 72 trials for the neutral condition.

#### Data analysis

The first fixation proportions (FFPs), dwell times from eye tracking, and manual response times (RTs) were analyzed using data from the visual search trials with correct responses. The FFPs were computed by assessing the percentage of fixations falling within a virtual circular area, having a radius of 2° centered around the stimuli of interest, encompassing both memory-matched and contrast distractors. The calculation of FFPs followed the criteria outlined by Zhang et al. [32], which involved considering only the first saccadic eye movement that was time-locked to the onset of the visual search array. Trials were excluded if the actual first fixations did not fall within the region of central fixation (2° around the central fixation) or if the duration of the first eye movement was less than 100 ms or greater than 500 ms. The capture effects on FFPs in each perceptual similarity condition were assessed by comparing the FFPs between memory-matched distractors in each perceptual similarity condition and the contrast distractors in the neutral condition. Attentional capture effects of RTs are calculated by the RTs cost of each perceptual similarity condition compared to the neutral condition. Dwell times, which represent the total duration of fixations within a specific region, were used to estimate the processing time required to evaluate the distractor once it had been initially selected [35, 36].

To assess the capture effect, we calculated the average performance on all neutral trials across all blocks and used this as a baseline for comparing performances across different levels of perceptual similarity. Subsequently, we categorized the trials into four matching types: exact, high-similar, low-similar, and neutral. All data were submitted to a one-way repeated-measures ANOVA with the factor of Matching Type, incorporating Greenhouse-Geisser corrections for violations of sphericity assumptions. Bonferroni adjustments were applied to pairwise comparisons to control for Type I error.

### Results and discussions

#### Memory test performance

The memory accuracy was assessed by the mean standard deviation (MSD) of the absolute deviation in the distance, measured in degrees of feature space, between the memory report and the actual value. The results from the repeated-measures ANOVAs revealed that the main effect of Matching Type were not statistically significant for both MSD, *F*(1.69, 33.71) = 2.92, *p* = 0.076, η_p_^2^ = 0.128, and RTs, *F*(1.83, 36.67) = 0.28, *p =* 0.736, η_p_^2^ = 0.014. These results suggested that neither the accuracy nor the recognition speed of the memory test was affected by the Matching Type.

#### Accuracy (ACC) and RTs for the visual search task

ACC for the visual search was 99.01%, and had no differences between conditions, *F*(3, 60) = 0.8, *p* = 0.5, η_p_^2^ = 0.038.

**Fig. 2:**
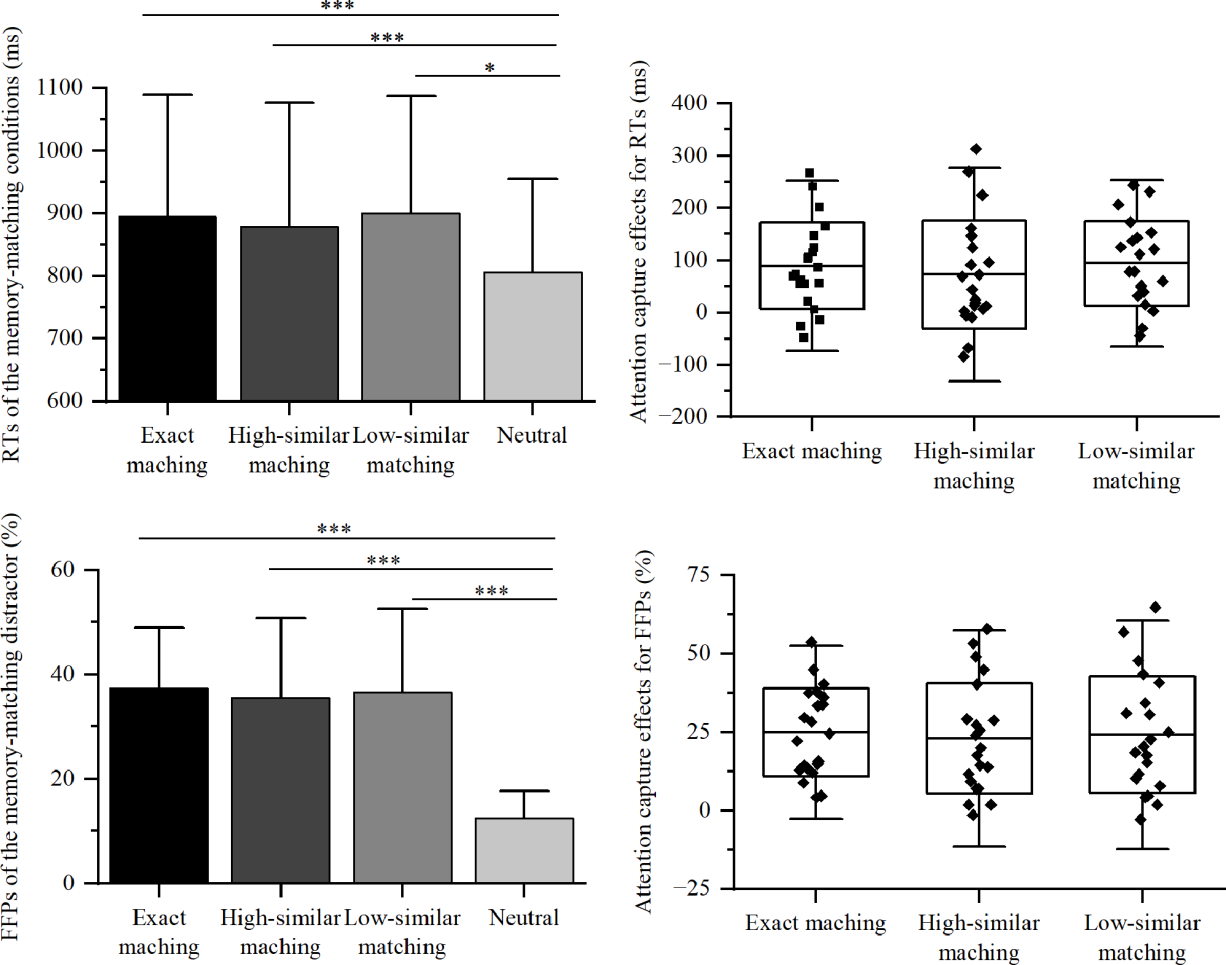
The FFPs, RTs, and attentional capture effects indexing by RTs (top panel) and FFPs (bottom panel) in each condition for Experiment 1. Each dot represents the attentional capture effect for one participant under a specific condition. Error bars represent the standard deviation of the mean. “*” represent *p* < 0.05, “***” represent *p* < 0.001

A total of 0.99% of incorrect response trials and 2.41% of outlier trials with RTs exceeding 3 SDs of each participant’s mean RTs were excluded from the data analysis. A repeated-measures ANOVAs for RTs revealed a significant main effect of Matching Type, *F*(3, 60) = 7.08, *p* < 0.001, η_p_^2^ = 0.261. The RTs for exact, high-similar, and low-similar matching conditions were comparable (895 ms, 878 ms, 900 ms; *ps* ≈ 1), yet significantly higher than those in the neutral condition (805 ms, *ps ≤* 0.027). As shown in Fig. 2, this indicated a robust but indistinguishable level of attentional capture across varying degrees of perceptual similarity (90 ms vs. 73 ms vs. 95 ms, *F*(2, 40) = 0.37, *p* = 0.696, η_p_^2^ = 0.018).

#### FFPs and dwell times for distractors

The ANOVA showed significant main effects for Matching Type on both FFPs, *F*(2.02, 40.47) = 29.97, *p* < 0.001, η_p_^2^ = 0.6, and dwell times, *F*(3, 60) = 8.04, *p* < 0.001, η_p_^2^ = 0.287. Post-hoc analyses revealed comparable FFPs and dwell times across exact, high-similar, and low-similar matching conditions (FFPs: 37.37% vs. 35.45% vs. 36.57%, *ps ≈* 1; dwell times: 237 ms vs. 227 ms vs. 240 ms, *ps ≈ 1*), but all significantly greater than the neutral condition (FFPs: 12.46%, *p ≤* 0.001; dwell time: 193 ms, *ps ≤* 0.066). As depicted in Fig. 2, no discernible differences were observed in the capture effects among the exact, high-similar, and low-similar matching conditions (24.91% vs. 22.99% vs. 24.11%, *ps ≈* 1), *F*(2, 40) = 0.32, *p* = 0.73, η_p_^2^ = 0.016.

The results of Experiment 1 demonstrated a robust attentional capture effect on both FFPs and RTs. Intriguingly, the extent of this capture effect remained unaffected by the perceptual similarity between memory items and distractors. This finding aligns with the hypothesis that attentional capture is primarily mediated by coarse rather than fine memory-distractor matching. In other words, it suggests that attentional capture is triggered when a distractor broadly resembles a WM item, regardless of the precise perceptual details of that resemblance.

## Experiment 2

### Methods

In Experiment 2, another twenty-one college students (7 males; age range: 18–25; mean age = 21.05) participated. The stimuli and visual search task were largely similar to those in Experiment 1, with a crucial difference that the memorized item was designed to match either the target (valid condition) or one of the distractors (invalid condition). Consequently, each of the three blocks (exact, high-similar, and low-similar matching) comprised 60 trials for each of the valid, invalid, and neutral conditions, totaling 180 trials per block.

### Results and discussions

All data were analyzed with repeated-measures ANOVAs with two within-subject factors of Matching Type (exact, high-similar, low-similar, neutral) and Validity **(**invalid, valid).

#### Memory test performance

The analysis of MSD results indicated no statistically significant main effects for Matching Type, *F*(1.56, 31.19) = 1.03, *p* = 0.388, η_p_^2^ = 0.049, and Validity, *F*(1, 20) = 1.07, *p* = 0.314, η_p_^2^ = 0.051. But their interaction were significant, *F*(2.26, 45.1) = 3.36, *p* = 0.038, η_p_^2^ = 0.144. The simple effect test revealed that MSD was significantly lower for the high-similar matching condition than the neutral condition (10.05° vs. 11.54°, *p* = 0.018) in valid trials. In addition, MSD was significantly lower for valid conditions than invalid conditions (9.23° vs. 11.34°, *p* = 0.023) in the exact matching condition. No other significant differences were found (*p ≥* 0.243). The results for RTs indicated significant main effects for Validity, *F*(1, 20) = 6.98, *p* = 0.016, η_p_^2^ = 0.259, but not for Matching Type, *F*(1.39, 27.82) = 0.1, *p* = 0.84, η_p_^2^ = 0.005, and their interaction, *F*(2.37, 47.32) = 1.67, *p* = 0.19, η_p_^2^ = 0.078. Specifically, the RTs in the valid condition were significantly shorter than the invalid condition (1779 ms vs. 1820 ms, *p* = 0.016).

#### ACC and RTs for the visual search task

The mean ACC was 99.35%. No main effects and interactions were found, *Fs* ≤ 2.79, *ps ≥* 0.11.

**Fig. 3:**
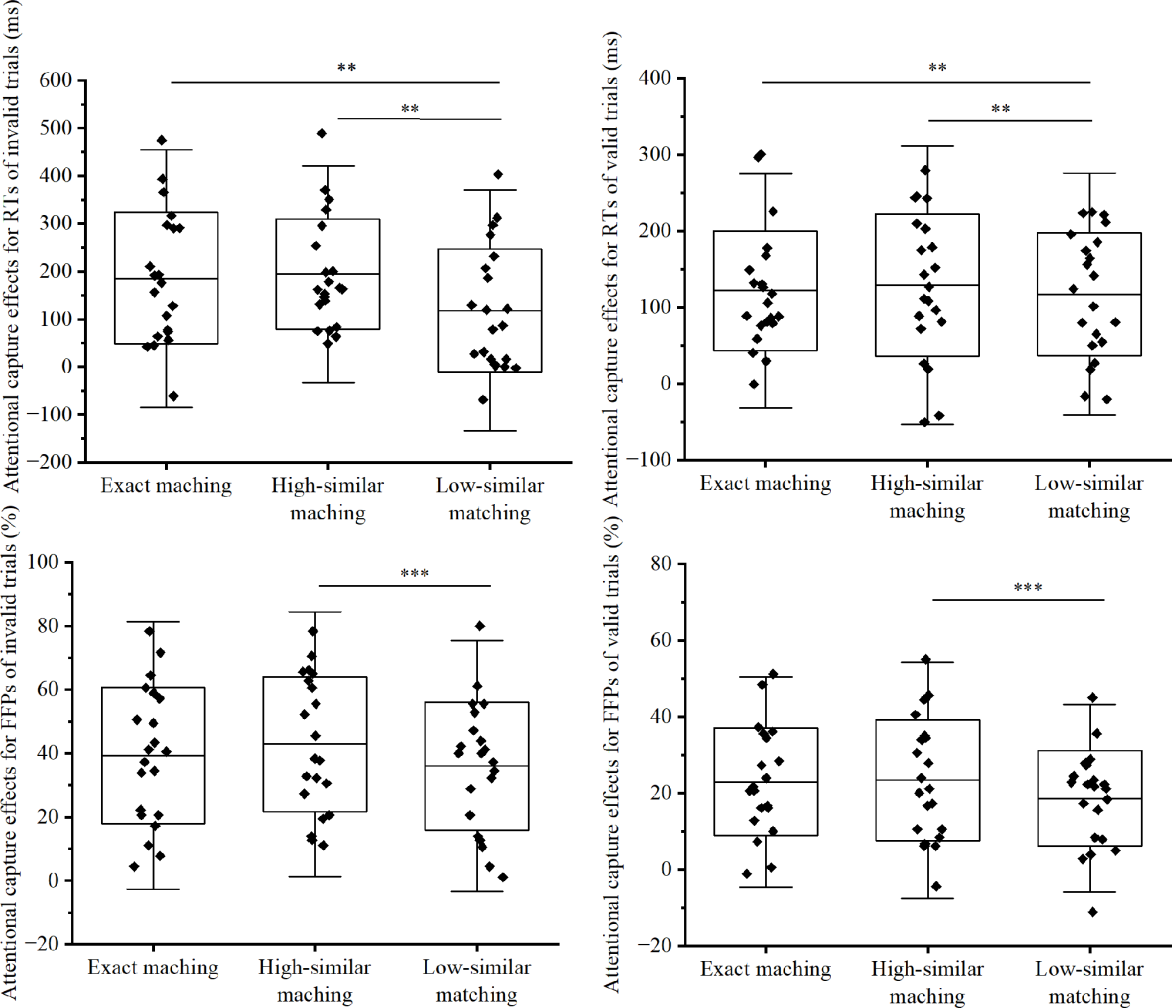
The attentional capture effects indexing by RTs (top panel) and FFPs (bottom panel) in each condition for Experiment 2. Each dot illustrates an individual’s attentional capture effect under a given condition, showing absolute costs from memory-matched distractors in invalid trials and benefits from memory-matched targets in valid trials. Error bars represent the standard deviation of the mean. “**” represent *p* < 0.01, “***” represent *p* < 0.001

For RTs, a total of 0.65% of incorrect response trials and 2.66% of outlier trials were excluded from the data analysis. The results of ANOVA revealed significant effects for Validity, *F*(1, 20) = 75.19, *p* < 0.001, η_p_^2^ = 0.79, and their interaction, *F*(1.92, 38.37) = 50.3, *p* < 0.001, η_p_^2^ = 0.716, but not for Matching Type, *F*(3, 60) = 2.04, *p* = 0.118, η_p_^2^ = 0.093. To further delve into the interaction effects, we independently calculated the RT benefits for valid trials by subtracting the RTs in the neutral condition from the RTs in each matching condition, and the RT costs for invalid trials by subtracting the RTs in each matching condition from the RTs in the neutral condition. One-sample t-tests indicated that all these capture effects were significantly greater than zero across all conditions (*ts* ≥ 4.21, *ps* < 0.001; see Fig. 3 for details). Subsequently, these capture effects were subjected to an ANOVA with two within-subject factors: Validity (valid vs. invalid) and Matching Type (exact, high-similar, low-similar). The results revealed significant effects for Validity, *F*(1, 20) = 4.8, *p* = 0.04, η_p_^2^ = 0.194, and Matching Type, *F*(1.55, 30.9) = 8.77, *p* = 0.002, η_p_^2^ = 0.305. In particular, the capture effect was stronger for invalid than valid trials (166 ms vs. 123 ms). However, the captures for exact and high-similar matching conditions were statistically indistinguishable (154 ms vs. 162 ms, *p ≈* 1), both significantly greater than low-similar matching (118 ms, *ps ≤* 0.002). No significant interactions were observed, *F*(2, 40) = 1.58, *p* = 0.219, η_p_^2^ = 0.073.

#### FFPs and dwell time for distractors

Due to the inclusion of valid trials, Experiment 2 used the search target from the neutral condition as the contrast stimuli for calculating capture effects in the valid condition. For invalid trials, the calculation method remained unchanged from Experiment 1. To streamline the statistical analysis, we determined the capture effects for each condition by subtracting the FFPs of the contrast stimuli from those of the memory-matched stimuli, as illustrated in Fig. 3. One-sample t-tests indicated all capture effects were significantly greater than zero, *ts* ≥ 6.78, *ps* < 0.001. Similar to RTs, the capture effects summited to ANOVA with factors of Matching Type and Validity. The results revealed significant effects for Validity (39.37% and 21.59% respectively for invalid and valid trials), *F*(1, 20) = 87.13, *p* < 0.001, η_p_^2^ = 0.813, and Matching Type, *F*(1.56, 31.19) = 4.83, *p* = 0.021, η_p_^2^ = 0.195, but not for their interaction, *F*(1, 20) = 0.74, *p* = 0.483, η_p_^2^ = 0.036. The capture effect for the high-similar matching (33.07%) was greater than the low-similar matching condition (27.28%, *p* < 0.001), but comparable with exact matching (31.09%, *p ≈* 1). The differences in capture effects between exact and low similar matching conditions were insignificant (*p* = 0.291).

We analyzed the dwell time for memory-matched stimuli compared to their respective contrast stimuli using an ANOVA with Validity and Matching Type as factors. The results indicated significant effects for Validity, *F*(1, 20) = 211.27, *p* < 0.001, η_p_^2^ = 0.914, Matching Type, *F*(3, 60) = 15.13, *p* < 0.001, η_p_^2^ = 0.431, and their interaction, *F*(3, 60) = 16.48, *p* < 0.001, η_p_^2^ = 0.452. Further analysis revealed no significant differences in dwell time across the various Matching Types (*ps* ≥ 0.448) for valid trials. However, for invalid trials, the dwell time for the neutral condition (203 ms) was significantly shorter than that for the exact (283 ms), high-similar (293 ms), and low-similar matching (271 ms) conditions (*ps* ≤ 0.001). Among the latter three conditions, only the high-similar and low-similar matching conditions exhibited a significant difference in dwell time (*p* = 0.006), whereas comparisons among all four matching conditions in valid trials did not reach statistical significance (*ps* ≥ 0.448).

Dwell times offer insights into the subsequent attention process following the initial selection [35]. For valid trials, dwell time primarily mirrors the efficiency of target identification, minimally influenced by the perceptual similarity of matches. Conversely, for invalid trials, it indicates the swiftness of disengaging from distractors once captured. The reactive reorienting hypothesis suggests that when WM representations consistently match distractors in task-irrelevant scenarios, these representations are encoded as negative templates. Although memory-matched distractors may initially capture attention, cognitive control mechanisms are triggered to promptly redirect attention away from such distractors [37]. In this context, the present results indicated that lower perceptual similarity can promote faster disengagement.

In summary, Experiment 2, in contrast to Experiment 1, intentionally introduced memory-matching stimuli that potentially shared features with the visual search target. Here, we observed a significant difference where perceptual similarity influenced the capture effects. Specifically, the high-similar matching condition exhibited a stronger attentional capture than the low-similar matching condition, implying the importance of task relevance.

## General discussion

While numerous studies have delved into attentional capture driven by irrelevant WM representations, the influence of perceptual similarity between memorized objects and visual search items remains poorly understood. The current study primarily focused on two questions about this issue: (1) whether fine or coarse memory-distractor matching was necessary for the occurrence of this capture? (2) whether the effect of perceptual similarity on memory-driven attentional capture was influenced by the task relevance? This issue pertains to whether the matching style, specifically whether it involves fine or coarse matching, shapes the impact of irrelevant WM representations on visual attention. The current study addressed these questions by deliberately manipulating three distinct levels of memory-distractor perceptual similarity in both task-irrelevant and task-relevant scenarios across two separate experiments. In Experiment 1, where WM representations only potentially matched features with distractors in the visual search (i.e., task-irrelevant scenario), a consistent lack of influence of perceptual similarity on memory-driven attentional capture was observed. This outcome supports the notion of a coarse matching assumption in these scenarios. However, when memorized objects were allowed to match features with the target (i.e., task-relevant scenario), a significant influence of perceptual similarity on attentional capture was found. This finding highlights the pivotal role of task relevance in shaping the matching style’s impact on memory-driven attentional capture.

Experiment 1 demonstrated comparable attentional capture effects across exact, high-similar, and low-similar representations, contradicting previous research conducted by Teng and Kravitz [23]. Their work suggested that the strength of memory-driven attentional capture hinges on the perceptual similarity between memory and distractor representations. As mentioned earlier in the introduction, our study differed significantly from Teng and Kravitz [23] in terms of the spectrum of perceptual similarity investigated. Our research concentrated on a narrower range of perceptual similarity while they examined the attentional capture disparities using notably more dissimilar feature values. Specifically, they compared memory-distractor distances of 15° or 35° to 75°, which is significantly distant from the internal memory representation, equivalent to dissimilar items [28]. Consequently, Teng and Kravitz [23] observed a gradual transition in attentional capture effects as the perceptual similarity between memory and distractor representations decreased, spanning from mentally similar to dissimilar representations. This methodological distinction and variation in feature value ranges could account for the divergence between their findings and the results of our current study.

The comparable attentional capture effects observed in Experiment 1 across exact, high-similar, and low-similar representations support the prevalent hypothesis that attentional guidance does not necessitate to match with precise, veridical memory features. Instead, the memory-matched item coarsely matched with the memory item still guides attention [2, 8, 12]. Yu et al. [38] posit that the target template fulfills distinct roles during visual search, guiding the initial attention allocation through a coarse, sensory-weighted process, while subsequent target identification necessitates a more precise representation for accurate decision-making. In Experiment 1, where the search target was an unequally gapped square, and the memorized color was neither a target nor used for target identification, we observed similar attentional capture effects using early eye-movement indices (e.g., FFPs). This suggests that irrelevant WM representations, regardless of their similarity to the target, interact with the initial attention guidance process in a coarse manner. These findings extend previous research by demonstrating that irrelevant WM representations, ranging from similar to nearly identical, exhibit equivalent attentional capture efficiency.

Although we did not directly manipulate the precision of WM representations, our findings, revealing similar attentional capture effects across a diverse range of WM representations, uphold the view that high-quality representations are not an indispensable requirement for memory-driven attentional capture [16, 29, 30]. However, our results contest the idea that memory quality unconditionally determines the extent of memory-driven attentional capture [19]. As mentioned in the introduction, task relevance could be a potential factor contributing to the divergence, as it differed from Experiment 1. In line with Williams et al. [19], when WM information became task-relevant in Experiment 2, we observed an enhanced attentional capture effect among both FFPs and RTs compared to Experiment 1. Critically, the enhancement in the capture effect was more pronounced for high-similar compared to low-similar representations. This finding indicates that task relevance not only amplifies the overall capture effect but also facilitates, to some extent, the modulation of attentional capture by perceptual similarity between memory and distractor items.

Task relevance significantly shapes not only the strategy utilized for memory-matched distractors, whether it be attention bias or rejection [14, 27, 39], but also the processing patterns of irrelevant WM representations in guiding visual attention selection. Notably, the target template consistently prioritizes cognitive resource allocation, relying on a highly precise internal representation [17, 28]. When the memorized feature gains relevance in facilitating the attentional selection of visual search targets in task-relevant scenarios, it initiates a cascading effect. This effect brings into play the unique characteristics of target template processing, which in turn influences the processing of irrelevant WM representations. This cascade results in a heightened competitive advantage for capturing attention [14, 40], further strengthened by an increased allocation of attention that enhances perceptual sensitivity. As a result, the demand for heightened perceptual precision within the internal WM representation becomes essential [2]. The intricate interplay between target template processing and the subsequent handling of irrelevant WM representations forms a complex interrelationship that profoundly impacts attentional capture.

Experiment 2 demonstrated modulation effects on RTs and FFPs with regard to attentional capture based on perceptual similarity. Our findings indicate that, to a certain extent, perceptual similarity impacts attentional capture even during the initial stages of visual search in task-relevant scenarios, though the FFP index may not fully capture the nuances of the earliest attentional stages. We failed to observe the decrease in the capture effect due to the suppression of similar information in the feature space surrounding WM items, as demonstrated by Kiyonaga and Egner [24]. We consider that the inconsistency may reflect the distinction between the target template and irrelevant representations in WM, although further research is needed to confirm this. Moreover, our observations consistently demonstrated similar attention capture effects in conditions involving both exact matches and highly similar matches across two experiments. This result may imply that the high-similar matching color remains within the range of exact matching in WM, making it challenging to perceptually differentiate between them during the process of capturing attention. Besides, our observation seems to contradict the dual-process framework proposed by Yu et al. [38] and Yu et al. [11] for target template-guided attention in visual search. This framework suggests that early attentional guidance relies primarily on a coarse sensory-driven process, with a more refined target representation becoming pivotal in later stages for efficient target identification. Given this discrepancy, two crucial points arise. Firstly, further investigation is warranted to elucidate the differences in how target templates and irrelevant WM representations steer visual attention in task-relevant contexts. Secondly, when formulating a theoretical framework to understand how internal WM representations guide visual attention, similar to the dual-process model for target templates [11, 38], it is imperative to factor in the influence of task relevance.

## Conclusions

In conclusion, our study provides evidence of how the perceptual similarity between WM representation and its matched distractor impacts attentional capture induced by these distractors. When WM representation is task-irrelevant, attentional capture occurs through coarse matching, independent of perceptual similarity. Conversely, when WM representation is task-relevant and serves as a target template, attentional capture is enhanced by higher perceptual similarity, suggesting that task relevance increases perceptual sensitivity to visual input. Our findings may imply distinct mechanisms underlying attentional capture triggered by irrelevant representations and target templates in WM. However, the current findings are primarily based on perceptual similarity in the feature of color, which exhibits a linear variation in feature space. Future research is necessary to investigate whether these results can be generalized to other diverse features, such as shape, orientation, or size.

## Electronic supplementary material

Below is the link to the electronic supplementary material.

Supplementary Material 1

## Data Availability

The data for all experiments are available at http://dx.doi.org/10.6084/m9.figshare.24151527.
